# Development of multiplex cross displacement amplification combined with lateral flow biosensor assay for detection of virulent *shigella sonnei*

**DOI:** 10.3389/fcimb.2022.1012105

**Published:** 2022-10-19

**Authors:** Yonglu Wang, Ziqiang He, Patigul Ablimit, Shunshi Ji, Dong Jin

**Affiliations:** ^1^ Ma’anshan Center for Disease Control and Prevention, Ma’anshan, China; ^2^ Pishan County Center for Disease Control and Prevention, Hotan Prefecture, China; ^3^ State Key Laboratory of Infectious Disease Prevention and Control, National Institute for Communicable Disease Control and Prevention, Chinese Center for Disease Control and Prevention, Beijing, China

**Keywords:** virulent shigella sonnei, multiplex cross displacement amplification, lateral flow biosensor, sensitivity, specificity

## Abstract

*Shigella sonnei* is the most common *Shigella* spp. in developed areas and the second most common in undeveloped regions. In this study, a multiple cross displacement amplification (MCDA) assay was used in combination with a lateral flow biosensor (LFB) assay to detect virulent *S. sonnei* strains containing the *ipaH* and *wbgX* genes. The multiplex MCDA-LFB assay detected *wbgX* at ≥1 pg/μL and *ipaH* at ≥10 fg/μL within 30 min in pure cultures maintained at 63°C. This assay was sensitive for ~37 CFU of virulent *S. sonnei* and ~3.7 CFU of *Shigella* spp. and enteroinvasive *E. coli* in stimulated fecal samples and had 100% specificity among 59 reference strains. The MCDA-LFB assay was also able to differentiate between virulent *S. sonnei* and other *Shigella* spp. and enteroinvasive *E. coli* among 99 clinical isolates. In summary, a multiplex MCDA-LFB assay was developed for rapid, convenient, point-of-care, and accurate identification of virulent *S. sonnei* within 30 min and at a constant temperature without the need for expensive lab equipment.

## Introduction

*Shigella* spp. is the major pathogen responsible for bacterial dysentery in both developed and developing countries, causing disease in approximately 190 million people and leading to 65,000 deaths per year (Kirk et al., 2015). Based on its biochemical and antigen characteristics, *Shigella* spp. is separated into four species/serogroups, *S. dysenteriae*, *S. flexneri*, *S. boydii*, and *S. sonnei*. *S. flexneri* and *S. sonnei* are the predominant *Shigella* spp. worldwide and *S. sonnei* is the main cause of shigellosis in developed areas, and the second most common in undeveloped regions (Thompson et al., 2015). Using 2004–2014 data from the National Infectious Disease Information Reporting System (NIDRS), 3,342,847 cases of bacillary dysentery were reported in China, with an average incidence rate of 22.89 cases per 100,000 person-years. *S. flexneri* was the most prevalent species followed by *S. sonnei* (2,191/6,278 isolates, 34.89%) (Chang et al., 2016).

Traditional *Shigella* spp. diagnosis is dependent on culture and serum agglutination, which are inefficient, expensive, and only able to detect a small proportion of actual shigellosis cases. The culture positivity rate of *Shigella* spp., which can cause infection with as few as 10–100 bacterial cells, depends on the number of living strains in food, fecal and environmental samples (Octavia and Lan, 2015). The conditions of sample collection and transportation, including temperature, pH, the use of antibiotics prior to specimen collection, and competition from other commensal organisms, can impact the success of a culture (Taylor and Schelhart, 1975; Vu et al., 2004). Molecular diagnostic methods including conventional, multiple, and real-time PCR have been developed to overcome the shortfalls of culture techniques (Gaudio et al., 1997; Vu et al., 2004; Lindsay et al., 2013). The gold standard method used to identify *Shigella* spp. is pure culture followed by serum agglutination. Slide agglutination using commercial antisera is hindered by the lack of high-quality sera, time constraints, and difficult-to-visualize results (Sun et al., 2011). Molecular methods such as multiplex PCR (Ojha et al., 2013) immunocapture PCR (Peng et al., 2002), matrix-assisted laser desorption/ionization-time of flight mass spectrometry (MALDI-TOF MS) assays, (Paauw et al., 2015) and high resolution melting (HRM) assay (Pakbin et al., 2022) were developed to differentiate between *Shigella* spp. While these methods allow rapid and high specificity identification of *Shigella*, they often require expensive equipment and involve complex methodologies. Our review revealed there was no isothermal amplification technique method for detection of *S. sonnei* and differentiation of virulent strains at the same time.

Multiple cross displacement amplification (MCDA) technique using strand displacement nucleic acid synthesis with the *Bst* polymerase reacts under isothermal conditions without the requirement of the expensive apparatus (Wang et al., 2015). All ten primers are designed to recognize ten distinct regions on the purpose sequence, enhancing the specificity and sensitivity of the MCDA reaction. Amplification products are a combination of various sequences of different fragment sizes, which can be detected by turbidimeters, colorimetric agents, gel electrophoresis, and nanoparticle lateral flow biosensor (LFB) (Wang et al., 2015). Dry-proof gold nanoparticle LFB is an easy-to-use method that reliably displays the results of amplification within a short time (Li and Macdonald, 2016). While migrating over the membrane, the amplification products encounter a series of reaction zones, which creates an output in these reaction areas (Li and Macdonald, 2016). Compared with other monitoring strategies such as turbidimeters, colorimetric agents, and gel electrophoresis, the critical advantage of LFB, is possible for the multiple identifications by detecting the amplification products labeled with different biomarkers (Li and Macdonald, 2016). MCDA combined with label-based LFB allows for the identification of multiple detections of target genes in one reaction (Wang et al., 2018). Multiplex MCDA-LFB methods are now used for the detection of several infectious agents, including *Salmonella* spp. (Wang et al., 2017), *Acinetobacter baumannii* (Hu et al., 2019), and SARS−CoV−2 (Luu et al., 2021).

The invasion plasmid antigen H (*ipaH*) gene, multiple copies of which are located on both the chromosome and plasmid, is widely used to detect *Shigella* spp. and enteroinvasive *Escherichia coli* (EIEC) (Hartman et al., 1990). The virulent *S. sonnei* strains (form I O polysaccharide, phase I) comprise a large virulence plasmid, which harbors both O polysaccharide and invasion (Xu et al., 2002). The *wbgX* gene encodes amino sugar synthetase and is located on the *S. sonnei* and *Plesiomonas shigelloides* O17 specific virulence plasmids with more than 99% sequence similar (Shepherd et al., 2000). In this study, a simple, visual, rapid, and sensitive method was developed to detect virulent *S. sonnei* using multiplex MCDA-LFB that was designed to target the *ipaH* and *wbgX* genes.

## Materials and methods

### Strains and preparation of DNA templates

A total of 161 strains including 122 *Shigella* spp., 11 enteroinvasive *E. coli* isolates, and 28 strains of other bacterial pathogen species were used to test the analytical specificity and application of the multiplex MCDA-LFB assays (**Table 1**). *Shigella* monovalent antisera (Denka Seiken, Japan) were used to serologically confirm the *S. sonnei* isolates. *S. flexneri *2a strain 301, *S. sonnei* CMCC 51081 (phase I), *S. sonnei* CMCC M6381 (phase II), and enterohemorrhagic *E. coli* EDL933 (ATCC 700927) were used to develop the conditions for the MCDA-LFB assay. The bacterial strains were cultured on Brain heart infusion agar or Columbia agar plates containing 5% defibrinated sheep blood and incubated in the ambient atmosphere or 5% CO_2_ atmosphere at 37°C for 18–24 hours. The bacterial genomic DNA was extracted using a QIAamp DNA Mini Kit (Qiagen, Germany) following the manufacturer’s instructions, and stored at -80°C until use. Qubit 4 Fluorometer (Thermo Fisher Scientific, USA) was used to quantify the genomic DNA. The genomic DNA of *S. sonnei* CMCC 51081was adjusted to 10 ng/μL using AE buffer (Qiagen, Germany) and then serially diluted to seven densities from 1 ng/μL to 1 fg/μL, including 100 pg/μL, 10 pg/μL, 1 pg/μL, 100 fg/μL, 10 fg/μL and 1 fg/μL. Three dilution series were prepared and then dilutions of the same concentrations were mixed to minimize errors during dilution.

**Table 1:** Strains used in this study and the results of MCDA assays.

Bacteria	Strain no./source^a^	No. of strains	MCDA LFB results^d^	
			*ipaH*	*wbgX*
*Shigella sonnei* phase I	CMCC 51081	1	+	+
*S. sonnei* phase I	Isolated strain	32	+	+
*S. sonnei* phase II	CMCC M6381	1	+	−
*S. sonnei* phase II	Isolated strain	25	+	−
*S. flexneri*	301	1	+	−
*S. flexneri*	Isolated strain	32	+	−
*S. dysenteriae*	CMCC^b^	12	+	−
*S. boydii*	CMCC^c^	18	+	−
enteroinvasive *E. coli*	CMCC 44825	1	+	−
enteroinvasive *E. coli*	Isolated strain	10	+	−
enterohemorrhagic *E. coli* O157:H7	EDL933 (ATCC 700927)	1	−	−
enteroaggregative *E. coli*	O42	1	−	−
enteropathogenic *E. coli*	2348/69	1	−	−
enterotoxigenic *E. coli*	10407	1	−	−
uropathogenic *E. coli*	UPEC 536	1	−	−
*Plesiomonas shigelloides*	CHPC 1.5797	1	−	−
*Listeria monocytogenes*	ATCC BAA-679	1	−	−
*Salmonella enterica* serovar Paratyphi A	CMCC 50001	1	−	−
*S. enterica* serovar Paratyphi B	CMCC 50001	1	−	−
*S. enterica* serovar Choleraesuis	ATCC BAA-664	1	−	−
*Enterobacter cloacae*	ATCC 700323	1	−	−
*Vibrio parahemolyticus*	ATCC 17802	1	−	−
*Yersinia enterocolitica*	Isolated strain	1	−	−
*Citrobacter freundii*	ATCC 8090	1	−	−
*Pseudomonas aeruginosa*	ATCC 27853	1	−	−
*Acinetobacter baumannii*	ATCC 17978	1	−	−
*Enterococcus faecium*	CGMCC1.2135	1	−	−
*E. faecalis*	CGMCC1.2136	1	−	−
*E. durans*	CGMCC 1.2284	1	−	−
*E. avium*	CGMCC 1.2505	1	−	−
*E. hirae*	CGMCC 1.2140	1	−	−
*E. mundtii*	CGMCC 1.2486	1	−	−
*Staphylococcus aureus subsp. aureus*	ATCC 25923	1	−	−
*Streptococcus pneumoniae*	ATCC 49619	1	−	−
*S. alactolyticus*	ATCC 43077	1	−	−
*S. pyogenes*	ATCC 19615	1	−	−
*Stenotrophomonas maltophilia*	CGMCC 1.1788	1	−	−
*Burkholderia cepacia*	CGMCC 1.1813	1	−	−

^a^CMCC, National Center for Medical Culture Collections; ATCC, American Type Culture Collection; CGMCC, China General Microbiological Culture Collection Center; CPHC, National Pathogen Resource.

^b^containing all 12 serotypes.

^c^containing all 18 serotypes.

^d^+, positive; −, negative.

### Design of the primers

The *ipaH* (GenBank accession no. M32063) and *wbgX* (GenBank accession no. AF285971) genes were used as targets to design the MCDA primers using Primer Primer 6.0 software and PrimerExploer V4 (Eiken Chemical Co., Ltd., Japan). Each set of primers, including two cross primers (CP1 and CP2), two displacement primers (F1 and F2), and six amplification primers (C1, C2, D1, D2, R1, and R2) was designed to recognize 10 distinct regions of each target gene. The C1 and D1 primes were labeled at 5’ end for LBF detection (Wang et al., 2017). Primer specificity was assessed using the National Center for Biotechnology Information (NCBI) BLAST. The primer sequences and the modification of optimal primers are listed in **Table 2**, and the position of the primers on the target genes is shown in **Supplemental Figure 1**. The oligomers were synthesized and purified using Tsingke Biological Technology (Tsingke, China).

**Table 2:** Primers used in this study.

Gene	Primers	Sequence and modification (5’-3’)	Length
*ipaH*	Ipa-F1	GGCTGGAAAAACTCAGTGC	19
	Ipa-F2	TGACTTTATCCCGGGCAAT	19
	Ipa-CP1	CATGTGAGCGCGACACGGTCAGCAGTCTTTCGCTGTTG	39
	Ipa-C1*	FITC-CATGTGAGCGCGACACGGTC	25
	Ipa-CP2	CCTTTTCGATAATGATACCGGCGCTCCTCCAGAATTTCGAGGC	44
	Ipa-C2	CCTTTTCGATAATGATACCGGCGC	24
	Ipa-D1*	Biotin-CTCAGTGGCATCAGCA	23
	Ipa-D2	TCTGCTCTCCCTGGGCA	17
	Ipa-R1	GGTTTTCCGGAGATTGTTC	19
	Ipa-R2	GTCCATCAGGCATCAGA	17
*wbgX*	Wbg-F1	GCAATCGGTATTCATCAACTT	21
	Wbg-F2	CTTATGCAACGGTATAAAATGG	22
	Wbg-CP1	GTGGCAATTCTTTTAACGCATCATCAGAAAGATCGATGATTTTCAGAA	49
	Wbg-C1*	FITC-GTGGCAATTCTTTTAACGCATCATC	30
	Wbg-CP2	CTATCCGCTTAAAAACTGATTCGGC-ACAGAACAACCAATTCCAAG	46
	Wbg-C2	CTATCCGCTTAAAAACTGATTCGGC	25
	Wbg-D1	Dig-TTTTTGCCATTCGTTGACGT	32
	Wbg-D2	CGCGATGATTTTATTAAGAAG	21
	Wbg-R1	TAGGCCATTCAGGCAATTC	19
	Wbg-R2	GATATTCATGCTTGGCATCT	20

*Modification at 5′ end of the primers. a, nt, nucleotide mer; monomeric unit. FITC, fluorescein isothiocyanate; Dig, digoxigenin.

### The singlex MCDA reaction

The singlex MCDA reaction for the *ipaH* and *wbgX* genes is conducted as described previously (Wang et al., 2017). WarmStart Colorimetric LAMP 2X Master Mix (New England Biolabs Inc., USA) was used to carry out the MCDA reaction. The MCDA was performed using a 25 μL mixture containing 12.5 μL of 2x reaction mixture, a 2.2 μL primer mixture including 0.4 μM each of F1 and F2, 0.8 μM each of C1 and C2, R1 and R2, D1 and D2, 1.6 μM each of CP1 and CP2, and 1 μL of the DNA template. The mixture was incubated at 63°C for 60 min and then at 80°C for 5 min to terminate the reaction. The optimal MCDA reaction temperature was determined with five temperatures ranging from 61°C to 65°C (with 1°C interval). *S. flexneri *2a strain 301, *S. sonnei* CMCC 51081, and CMCC M6381 were used as the positive control, and enterohemorrhagic *E. coli* EDL933 was used as negative control for the *ipaH* gene. *S. sonnei* CMCC 51081 was used as the positive control, and *S. flexneri *2a strain 301, *S. sonnei* CMCC M6381, and enterohemorrhagic *E. coli* EDL933 were used as the negative control for the *wbgX* gene. The mixture without the DNA template was used as the blank control. The MCDA reaction was confirmed using four monitoring techniques, including macroscopic color change observed with a color change from pink to yellow, turbidimeters (LA-320C, Eiken Chemical Co., Ltd., Japan) with the turbidity of >0.1 as positive, and 2% agarose gel electrophoresis with specific ladder DNA amplicons as positive, and LFB detection. The lateral flow dipsticks (MGHD2, Milenia HybriDetect 2T, Germany) with three lines, two test lines and one control line, were used to detect the labeled MCDA amplicons. The 5’ end of the *ipaH* C1 and D1 primers was labeled with fluorescein isothiocyanate (FITC) and Biotin. The FITC and Biotin amplicons were captured by the first test line using a Biotin ligand. Red lines then appeared on the first test line (T L1). The FITC and Dig (digoxigenin) amplicons used to detect the *wbgX* gene were caught by the second test line (T L 2) which also turned red. The control line captured the anti-FITC antibody and was used to ensure that the LFB assay was functioning. For LFB detection, 0.5 μL MCDA products were added to the sample pad of the biosensor, followed by 80 μL of HybriDetect Assay Buffer. Results were observed after incubating the sample for 2–5 min at room temperature. The MCDA operations were performed with independent laboratory equipment and experimental consumables. Blank control was added in every batch of MCDA reactions and LFB detections to avoid contamination.

### MCDA assay sensitivity

The sensitivity of the MCDA-LFB assay for the *ipaH* and *wbgX* genes was respectively determined with 1 µL of serially diluted genomic DNA from *S. sonnei* CMCC 51081.The sensitivity of the MCDA-LFB assay in artificially contaminated human feces was determined by inoculating *S. sonnei* CMCC 51081 into the brain-heart infusion broth and cultivating overnight. Strain solutions (50 μL) were transferred to 5 ml brain-heart infusion broth and incubated to OD_610_ 0.61 with agitation, which was equivalent to 3.7×10^8^ CFU (colony-forming units)/ml using the plate count method. The cultures were then 10x serially diluted from 10^7^–10^1^ CFU per ml using normal saline. Seven concentrations of *S. sonnei* CMCC 51081 were mixed with human stool samples from healthy volunteers. The *S. sonnei* CMCC 51081 DNA template in spiked stool was extracted as previously described (Song et al., 2005). The experiment was independently performed in triplicate, 100 μL AE buffer (Qiagen, Germany) was used to elute the extracted DNA, and 1 μL of each supernatant was used for MCDA detection.

### Optimization of multiplex MCDA primer concentration

The concentration ratio of the two sets of *ipaH* and *wbgX* primers was adjusted to ensure the simultaneous amplification of both genes according to their sensitivity. The total primer amount was fixed at 2.2 μL in a 25 μL MCDA reaction. Primer mixtures of 10 μL each of F1 and F2, 20 μL each of C1 and C2, R1 and R2, D1 and D2, and 40 μL each of CP1 and CP2 were used to make a 100μM concentration of the two genes. The five concentration ratios included 0.2–1.0 μL of the *ipaH* gene primer mix and 2.0–1.2 μL of the *wbgX* gene primer mix with a 0.2 μL interval. Reaction results were detected using the LFB assay.

### MCDA assay specificity

The multiplex MCDA reactions were performed with different DNA templates from 59 reference strains including 12 *S. dysenteriae*, 18 *S. boydii*, one enteroinvasive *E. coli*, and 28 other bacterial pathogens (**Table 1**) using the conditions described above to evaluate MCDA assay specificity. Each DNA template was independently analyzed in triplicate.

### Use of the MCDA-LBF assay on clinical isolates

Clinical *Shigella* spp. isolates including 32 *S. sonnei* phase I, 25 *S. sonnei* phase II, 32 *S. flexneri*, and 10 enteroinvasive *E. coli* were used to evaluate the clinical applicability of the MCDA-LBF assay. The clinical isolates were identified using a biochemical test with API 20E (BioMérieux, France) and slide agglutination. A single colony was placed into a 1.5 ml microcentrifuge tube with 100 mL distilled water. The sample was cooked for 10 min at 100°C before cooling for 5 min on ice. The supernatant was transferred to a new 1.5 ml microcentrifuge tube, centrifuged at 12,470 g for 10 min, and used as a template to perform the multiplex MCDA-LFB assay.

### Ethics statements

The study was performed following the guidelines of the Declaration of Helsinki and approved by the Ethics Committee of National Institute for Communicable Disease Control and Prevention, China CDC (No. ICDC2017-003).

## Results

### Functionality of the MCDA assay

**Figure 1:**
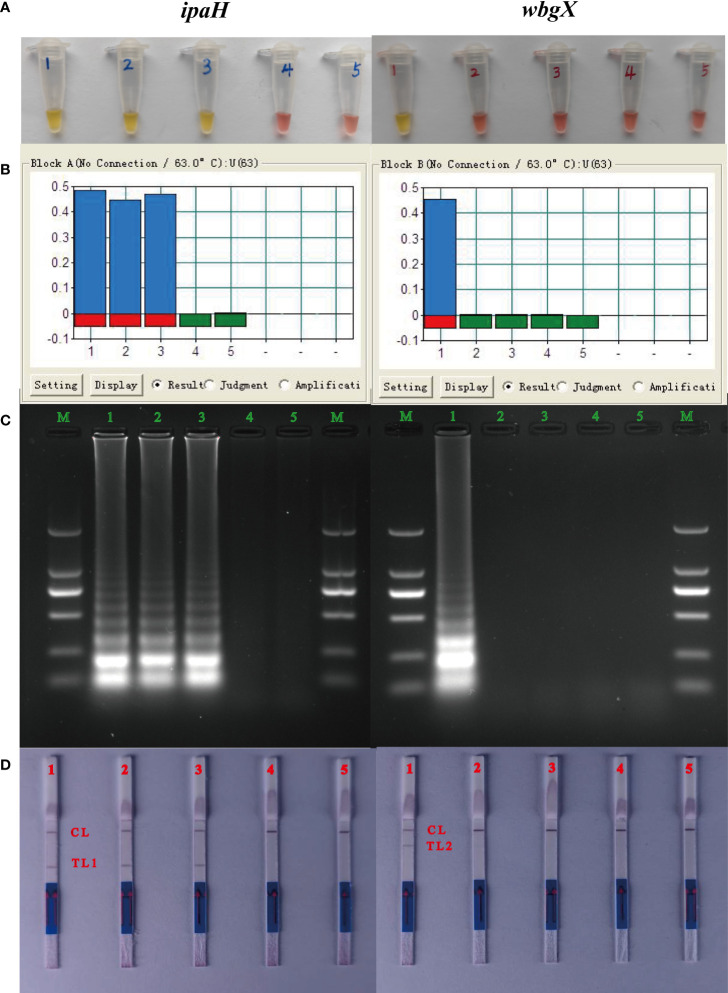
Verification of the MCDA products of *ipaH* and *wbgX* genes. Tubes/lanes/strips/biosensors 1-5: 1, *S. sonnei* CMCC 51081 (phase I); 2, *S. flexneri* 301; 3, *S. sonnei* CMCC M6381 (phase II); 4, enterohemorrhagic *E. coli* EDL933 (Negative control); 5, distilled water (Blank control). **(A)** Reaction products of the MCDA assay of *ipaH* and *wbgX* genes were visually detected by color change. **(B)** Turbidity in real-time using the Loopamp Real-time Turbidimeter LA-320C for the MCDA assay. **(C)** 2% agarose gel electrophoresis results of the MCDA assay. **(D)** LFB was applied for visual detection of the MCDA assay, CL (control line), T L1 (test line 1), and T L2 (test line 2). For *ipaH* gene, positive results were observed with Tubes 1-3 with the color changed from pink to yellow, lanes 1-3 with >0.1 turbidity changes with turbidimeters, strips 1-3 with the ladder DNA amplicons, and biosensors 1-3 with the red test line 1. Negative results were observed with Tubes/lanes/strips/biosensors 4-5 with negative control and blank control. For *wbgX* gene, positive results were observed with Tubes/lanes/strips 1 and biosensors 1 with the red test line 2, Negative results were observed with Tubes/lanes/strips/biosensors 4-5.

To demonstrate the functionality of the MCDA assay, genomic DNA from *S. flexneri *2a strain 301, *S. sonnei* CMCC 51081, *S. sonnei* CMCC M6381, and enterohemorrhagic *E. coli* EDL933 was amplified for 60 min at 63°C using the *ipaH* and *wbgX* primers. Distilled water was used as a blank control. The color change from pink to yellow (**Figure 1A**), >0.1 change in turbidity (**Figure 1B**), presence of ladder DNA amplicons (**Figure 1C**), and the red test lines 1 and 2 for the *ipaH* and *wbgX* genes, respectively, were observed macroscopically. *S. flexneri *2a strain 301, *S. sonnei* CMCC 51081, and *S. sonnei* CMCC M6381 were positive for the *ipaH* gene while enterohemorrhagic *E. coli* EDL933 and the blank control were negative. *S. sonnei* CMCC 51081 was positive for the *wbgX* gene while *S. flexneri *2a strain 301, *S. sonnei* CMCC M6381, *enterohemorrhagic E. coli* EDL933, and the blank control were negative (**Figure 1**). These results indicated that the *ipaH* and *wbgX* primers were appropriate candidates for developing a *S. sonnei*-specific multiplex MCDA-LFB assay.

### Determining the optimal temperature

Five temperatures from 61–65°C with a 1°C increment were used to determine the optimal MCDA temperature for the 60 min reaction and 10 pg *S. sonnei* CMCC 51081 DNA was used as a template. Positive results (>0.1 turbidities) were observed for the *ipaH* and *wbgX* genes within 20 min at all specified temperatures (**Supplemental Figure 2**). A 20 min amplification at 63°C and 5 min termination at 80°C were used for subsequent experiments.

### Sensitivity of the singlex MCDA assay for the *IpaH* and *WbgX* genes

**Figure 2:**
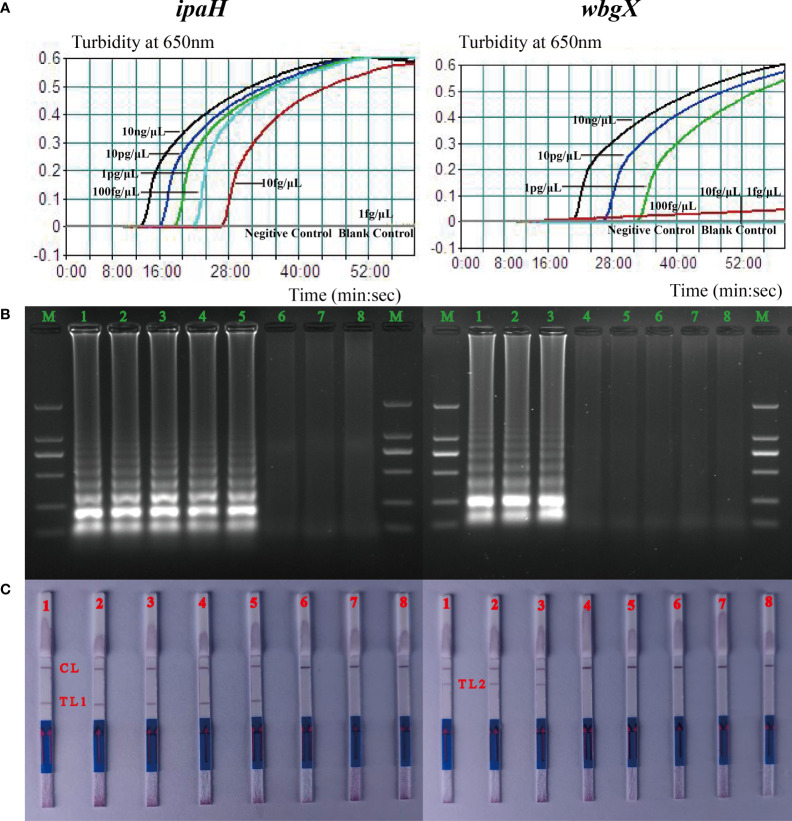
Detection limits of the *ipaH* and *wbgX* genes of the singlex MCDA-LFB assay. The six dilution templates of *S. sonnei* CMCC 51081 including 10 ng, 10 pg, 1 pg, 100 fg, 10 fg, and 1 fg were subjected to MCDA reactions to detect limits of *ipaH* and *wbgX* genes. **(A)** Real-time sensitivity of the singlex MCDA assay of two genes monitored by measuring the turbidity (optimal density at 650 nm). The turbidity >0.1 was considered positive for the two genes. The detection limits were 10 fg/μL for *ipaH* and 1 pg/μL for *wbgX* genes. **(B)** Gel electrophoresis was applied for analysis of the sensitivity of *ipaH* and *wbgX* genes. Strips M, DNA marker DL2000; Strips 1–6, PCR results for six dilutions; Strips 7, negative control; Strips 8, blank control. In agarose gel analysis of the two genes, the MCDA products showed the typical ladder-like pattern amplicons, and the detection limit matched the turbidity data. **(C)** LFB applied for analysis of *ipaH* and *wbgX* genes MCDA products. Biosensors 1–6, MCDA results for six dilutions; Biosensor 7, negative control; Biosensor 8, blank control. CL (control line), T L1 (test line 1), T L2 (test line 2). The positive results were observed at Biosensor 5 with the red line of T L1 and Biosensor 3 with the red line of T L2 which meant the detection limits of the *ipaH* gene was 10 fg/μL for and *wbgX* gene was 1 pg/μL. The detection limits matched the turbidity data and gel electrophoresis results.

Sensitivity of the singlex MCDA assay for virulent *S. sonnei* was evaluated by analyzing the products yielded from the diluted *S. sonnei* CMCC 51081 DNA templates (10 ng/μL, 10 pg/μL, 1 pg/μL, 100 fg/μL, 10 fg/μL and 1 fg/μL) using the method previously described. Enterohemorrhagic *E. coli* EDL933 served as the negative control and the mixture without the DNA template was used as a blank control. The detection limits were 10 fg/μL for *ipaH* and 1 pg/μL for *wbgX* (**Figure 2A**). The MCDA products were analyzed on a 2% agarose gel by electrophoresis and LFB and the results were consistent with the turbidity curves (**Figures 2B, C**).

### Optimization of the multiplex MCDA-LBF primer concentration

**Figure 3:**
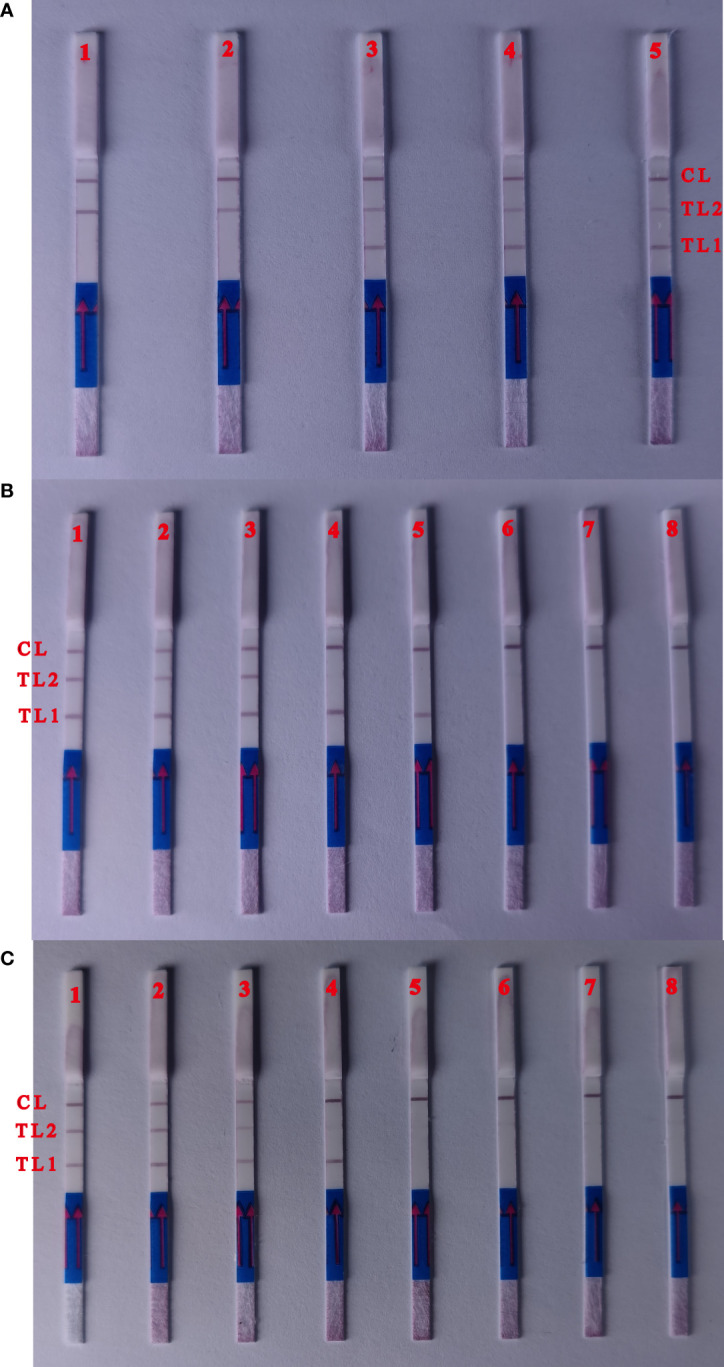
Optimization of the ratio of two primers and detection limit of multiple targets MCDA assay. **(A)** Biosensors 1, the primer concentration with 0.2 μL *ipaH* gene primers mix and 2.0 with μL *wbgX* gene primers mix; biosensors 2, 0.4 μL (*ipaH* gene) and 1.8 μL (*wbgX* gene); biosensors 3, 0.6 μL (*ipaH* gene) and 1.6 μL (*wbgX* gene); biosensors 4, 0.8 μL (*ipaH* gene) and 1.4 μL (*wbgX* gene); biosensors 5 1.0 μL (*ipaH* gene) and 1.4 μL (*wbgX* gene). Two red test lines appeared from the primer concentrations with 0.6μL *ipaH* gene 1.6 μL *wbgX* gene primers mixtures. **(B)** The sensitivity of multiple MCDA-LFB reactions with the serial dilutions genomic DNA of *S. sonnei* CMCC 51081. Biosensors 1–6, multiple MCDA results for six dilutions including 10 ng, 10 pg, 1 pg, 100 fg, 10 fg, and 1 fg; Biosensor 7, negative control; Biosensor 8, blank control. The sensitivity of multiple MCDA reactions was 1 pg/μL (positive results of Biosensors 3). The positive results could observe at Biosensors 5 with the concentration of 10 fg/μL with *ipaH* gene in the multiple MCDA reactions. No red test lines could be observed with negative control and blank control. **(C)** The sensitivity of the multiplex MCDA assay in artificially contaminated human feces samples. Biosensors 1–6: *S. sonnei* CMCC 51081 strain at 3700 CFU, 370 CFU, 37 CFU, 3.7 CFU, 0.37 CFU and 0.037 CFU per reaction; Biosensor 7, negative control; Biosensor 8, blank control. The positive results were detected for samples containing 37 CFU per reaction, and the red test lines of *ipaH* gene appeared in the sample with 3.7 CFU.

The MCDA primer concentrations were adjusted to promote simultaneous amplification of the reaction and ensure the production of reliable multiplex MCDA products given differences in the detection limit of *ipaH* and *wbgX*. Two red test lines were observed using 1.6 μL *wbgX* and 0.6μL *ipaH* primer mixtures. These concentrations were selected for the multiplex MCDA-LFB reaction conditions (**Figure 3A**).

### The sensitivity and specificity of the multiple MCDA-LBF assay

The sensitivity of the multiplex MCDA reaction was 1 pg/μL using serial dilutions of *S. sonnei* CMCC 51081 genomic DNA (**Figure 3B**). Positive results were observed using 10 fg/μL of the *ipaH* gene in a 25 min multiplex MCDA reaction. Only the control line was observed using *E. coli* EDL933 and the negative control. Positive results were detected for artificially contaminated human feces samples containing ≥3.7×10^3^ CFU per ml, and the red test line representing the *ipaH* gene appeared in the sample containing 3.7×10^2^ CFU (**Figure 3C**), indicating that the multiplex MCDA assay sensitivity was ~37 CFU for the *wbgX* gene and ~3.7 CFU for the *ipaH* gene.

To determine multiplex MCDA specificity, the assay was performed with DNA templates from 30 *Shigella* spp. reference strains including 12 *S. dysenteriae*, 18 *S. boydii*, one enteroinvasive *E. coli* reference strain, and 28 other bacterial pathogen species (**Table 1**). Positive results of the *ipaH* gene and negative results of the *wbgX* gene were observed using 31 reference strains including 30 *Shigella* spp. strains and one enteroinvasive *E. coli* strain. Negative results of both the *ipaH* and *wbgX* genes were observed using 28 strains from other bacterial genera.

### Application of the MCDA-LBF assay in clinical cultures

A total of 99 clinical isolates including 32 *S. sonnei* phase I, 25 *S. sonnei* phase II, 32 *S. flexneri*, and 10 enteroinvasive *E. coli* were used to evaluate the applicability of the MCDA-LBF assay. The *Shigella* spp. strains were identified using slide agglutination. Positive results of the *ipaH* gene were acquired using all isolates, while positive results of the *wbgX* gene were only observed using *S. sonnei* phase I.

## Discussion

The multiplex MCDA-LFB assay has several advantages over other molecular methods including multiplex PCR, real-time PCR, and high-resolution melting (HRM) used to identify *Shigella* spp. First, all reactions can be performed under isothermal conditions and positive amplification can be directly observed macroscopically, thus eliminating the use of large and complex instruments required for PCR-based systems. Second, amplification efficiency is extremely high, and many products can be obtained within 25 min. Third, the reaction uses multiple primers that recognize distinct regions of the target gene, thus making it highly sensitive and specific. Finally, combined with LFB, the MCDA assay achieves multiple target applications, which increases the possible uses of this method (Wang et al., 2017). As a result, the MCDA-LFB assay is widely used for nucleic acid analysis because of its simplicity, rapidity, high efficiency, and specificity (Hu et al., 2019; Luu et al., 2021). The current study developed a multiplex MCDA-LFB assay that was able to sensitively and accurately identify virulent *S. sonnei* using the *ipaH* gene and O-antigen synthesis gene, *wbgX*.

A reaction temperature of 63°C was chosen to simplify the experimental conditions and allow the two target genes to react synchronously. At this temperature, two gene reactions could be completed in 25 min, allowing for the rapid detection of virulent *S. sonnei*. The singlex MCDA assay was able to detect DNA samples at concentrations as low as 10 fg/μL for the *ipaH* gene and 1 pg/μL for the *wbgX* gene. Because of the difference in the copy numbers, the sensitivity of the *ipaH* gene with MCDA assay was much high than the *wbgX* gene. Due to differences in the detection limits of the two genes, five primer concentrations were adjusted to achieve simultaneous amplification during the reaction and 1.6 μL *wbgX* gene and 0.6μL *ipaH* gene primer mixtures were chosen for the multiplex MCDA-LFB reaction conditions. Using this primer concentration, 1 pg/μL of the *wbgX* gene and 10 fg/μL of the *ipaH* gene from virulent *S. sonnei* genomic DNA was detected in the multiplex MCDA reaction after 25 min which was consistent with the singlex MCDA-LFB assay. This multiplex MCDA assay could detect as few as ~37 CFU of virulent *S. sonnei* and ~3.7 CFU of *Shigella* spp. in artificially contaminated human feces samples. The sensitivity of this MCDA-LFP assay was higher than other isothermal amplification methods such as the LAMP assay and considerably better than PCR at detecting the *ipaH* gene from *Shigella* spp. (Song et al., 2005). Positive results from spiked feces samples indicated that the multiplex MCDA-LFB could perform well using clinical samples. The analytical specificity of the multiplex MCDA-LFB assay was demonstrated for 30 *Shigella* spp. reference strains, one enteroinvasive *E. coli* strain, and 28 other bacterial pathogen species. This multiplex MCDA-LFB assay showed high specificity for *Shigella* spp. and enteroinvasive *E. coli* strains, as well as other reference strains. To determine whether the assay could be applied to clinical isolates, boiled DNA from 57 *S. sonnei* strains, including 32 phase I and 25 phase II strains, and 42 *S. flexneri* and enteroinvasive *E. coli* strains were analyzed. The MCDA-LFB method was able to differentiate between *S. sonnei* and other *Shigella* spp. and enteroinvasive *E. coli*. The detection time was only 30 min, including 20 min for amplification, 5 min for termination the reaction, and 5 min for detection.

In summary, a multiplex MCDA-LFB assay was developed for the rapid (30 min), convenient, and accurate identification of virulent *S. sonnei* at a constant temperature without the use of expensive equipment. This assay could also be used to detect *Shigella* spp. and enteroinvasive *E. coli*. The multiplex MCDA-LFB assay could promote the rapid and reliable clinical diagnosis and epidemiological investigation of virulent *S. sonnei* in the field, during point-of-care, and on-site in various laboratories including resource-challenged settings.

## Data availability statement

The original contributions presented in the study are included in the article/**Supplementary Material**. Further inquiries can be directed to the corresponding author.

## Ethics statement

The studies involving human participants were reviewed and approved by The Ethics Committee of National Institute for Communicable Disease Control and Prevention. Written informed consent for participation was not required for this study in accordance with the national legislation and the institutional requirements.

## Author contributions

Conceptualization, DJ. Methodology, YW, ZH, and PA. Data curation, YW, SJ, and DJ. Writing—review and editing, SJ, and DJ. All authors contributed to the article and approved the submitted version.

## Funding

This work was supported by 2015011115 from Basic Research Project of Shanxi Province and 2018RU010 from Units of Discovery of Unknown Bacteria and Function.

## Conflict of interest

The reviewer LG declared a shared affiliation with the authors to the handling editor at the time of review.

The remaining authors declare that the research was conducted in the absence of any commercial or financial relationships that could be constructed as a potential conflict of interest.

## Publisher’s note

All claims expressed in this article are solely those of the authors and do not necessarily represent those of their affiliated organizations, or those of the publisher, the editors and the reviewers. Any product that may be evaluated in this article, or claim that may be made by its manufacturer, is not guaranteed or endorsed by the publisher.
