# Supplementary material for: Development of multiplex cross displacement amplification combined with lateral flow biosensor assay for detection of virulent shigella sonnei
Source: Front Cell Infect Microbiol. 2022 Oct 19;12:1012105. doi: 10.3389/fcimb.2022.1012105 (PMC9627043; doi:10.3389/fcimb.2022.1012105)
Supplement: Supplementary file 1 [file DataSheet_1.pdf]

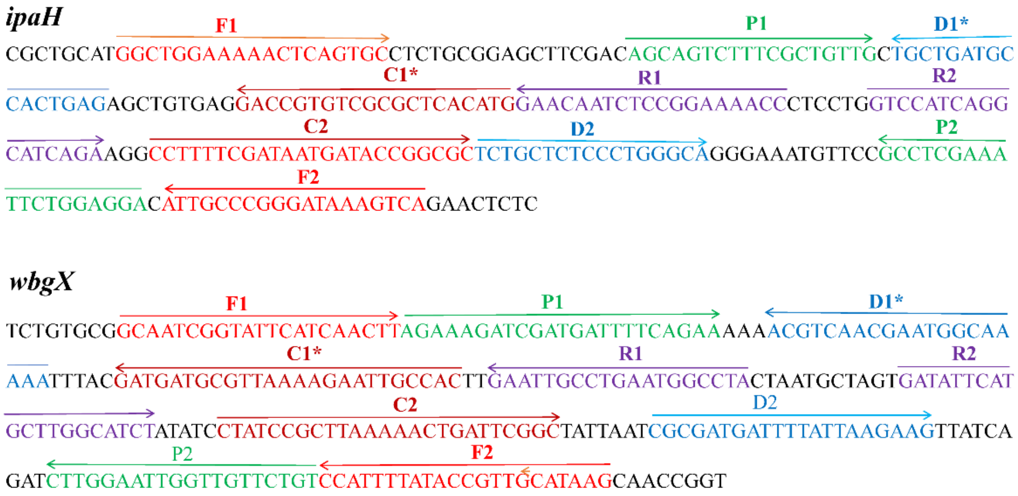

Supplemental Figure 1 The MCDA primers' locations and sequences of *ipaH* and *wbgX* genes.

Nucleotide sequences of the sense strands of two genes are shown. Primer CP1 consisted of the C1 direct sequence and the P1 complementary sequence. Primer CP2 consisted of the C2 complementary sequence and the P2 direct sequence. The primer sequences are highlighted with different colors. The right and left arrows indicate sense and complementary sequences. *, Modification at 5′ end of the primers.

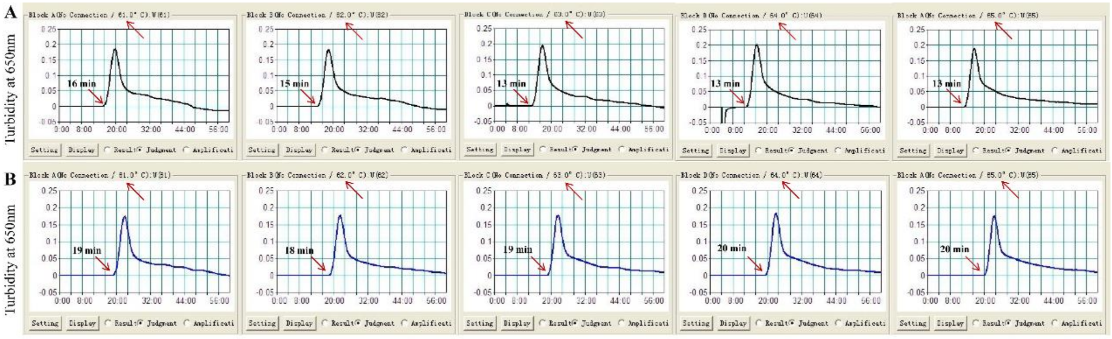

Supplemental Figure 2 The optimal amplification temperature for MCDA reaction. The MCDA amplifications of *ipaH* gene (A) and *wbgX* gene (B) were monitored by the measurement of turbidity and the turbidity of >0.1 was considered as positive. Five kinetic graphs at five temperatures (61 °C-65 °C, 1 °C intervals) were generated with target DNA at the level of 10 pg per reaction. The reaction times were showed in the Figure.
